# Spontaneous Usage of Different Shortcuts Based on the Commutativity Principle

**DOI:** 10.1371/journal.pone.0074972

**Published:** 2013-09-23

**Authors:** Robert Gaschler, Bianca Vaterrodt, Peter A. Frensch, Alexandra Eichler, Hilde Haider

**Affiliations:** 1 Universität Koblenz-Landau, Koblenz, Germany; 2 Humboldt-Universität, Berlin, Germany; 3 Universität Köln, Köln, Germany; Utrecht University, Netherlands

## Abstract

Based on research on expertise a person can be said to possess integrated conceptual knowledge when she/he is able to *spontaneously* identify task relevant information in order to solve a problem efficiently. Despite the lack of instruction or explicit cueing, the person should be able to recognize which shortcut strategy can be applied – even when the task context differs from the one in which procedural knowledge about the shortcut was originally acquired. For mental arithmetic, first signs of such adaptive flexibility should develop already in primary school. The current study introduces a paper-and-pencil-based as well as an eyetracking-based approach to unobtrusively measure how students spot and apply (known) shortcut options in mental arithmetic. We investigated the development and the relation of the spontaneous use of two strategies derived from the mathematical concept of commutativity. Children from grade 2 to grade 7 and university students solved three-addends addition problems, which are rarely used in class. Some problems allowed the use of either of two commutativity-based shortcut strategies. Results suggest that from grade three onwards both of the shortcuts were used spontaneously and application of one shortcut correlated positively with application of the other. Rate of spontaneous usage was substantial but smaller than in an instructed variant. Eyetracking data suggested similar fixation patterns for spontaneous an instructed shortcut application. The data are consistent with the development of an integrated concept of the mathematical principle so that it can be spontaneously applied in different contexts and strategies.

## Introduction

Given the role of self-guided learning and performance in the development of mathematical abilities and concepts, some recent studies have focused on *spontaneous* recognition of mathematical aspects of situations [1–3]. In a similar vein, Verschaffel and colleagues [4] have called for helping students to become experts in flexibly selecting the most efficient strategy for the current task and social context. This often, in the first place, involves recognizing that there is an alternative option for solving the current arithmetic problem. As implied by the expertise metaphor, direct instruction or cueing concerning the most efficient strategy will necessarily often be lacking. Just as it is unreasonable to expect that an expert is being told which knowledge applies in the given situation, it is unreasonable to expect that students take full advantage of arithmetic knowledge unless they can recognize and exploit shortcut options without having to rely on instructions or direct cues. Therefore, education could profit from means to diagnose and develop spontaneous knowledge application. Accordingly, in the current study we introduce a paper and pencil-based as well as an eyetracking approach to unobtrusively assess spontaneous application of two shortcuts that are procedurally different but are both based on the concept of commutativity. Rather than studying the acquisition of new concepts or strategies, our focus was on factors that determine whether or not a person recognizes and applies a shortcut option without being instructed to do so. The advantage of such spontaneous shortcut application is that it might reveal different aspects of the quality of the person’s knowledge than those aspects that can be tapped by more direct testing.

We borrowed from research on expertise [5–10] that a person can be said to possess integrated procedural (i.e., strategy) and conceptual (i.e., boundary conditions) knowledge when she/he is able to spontaneously identify task relevant information allowing to solve a problem in a highly efficient way [11,12]. Involvement of conceptual knowledge is especially plausible if spontaneous recognition and usage of shortcuts takes place on task material that was not previously used to teach the shortcut. Furthermore, behavior should reveal links between shortcuts based on the same concept, even when they are considerably different in procedure. Based on previous research [13–16] we expected that with increasing age, participant should adaptively select the shortcut strategy that best fits the shortcut options of the current task material [4] and usage of different shortcuts that are based on the same mathematical principle should increasingly correlate with age.

We tested this conjecture for the mathematical principle of commutativity. Commutativity is one of the core concepts that children should develop during the first years of school. The commutativity principle concerns the addition or multiplication of two numbers: The order in which the numbers are added or multiplied does not affect the sum or product (*a* + *b* = *b* + *a*; *a* × *b* = *b* × *a*). Because commutativity is a fundamental property in arithmetic, it is not surprising that it has been a focus of research in mathematics education and cognitive psychology. The core property of commutativity, the order-irrelevance principle, is ubiquitous in everyday situations, at least in a non-numerical sense. Already toddlers can experience that some activities follow the order-irrelevance principle while others do not. For example, putting on a pair of socks follows the order-irrelevance principle; that is, it does not matter in which order you put on your socks. By contrast, putting on underpants and trousers clearly does require a strict order. Thus, in everyday situations children have lots of opportunities to learn that order is relevant for some activities but irrelevant for others.

Consequently, several studies on commutativity have shown that children have at least some understanding of the concept of commutativity before entering school [16–20]. First graders seem to have at least some understanding of commutativity when adding numbers [21]. Yet, it is less clear whether this involves understanding the reasons for why the order is irrelevant when adding numbers or just the fact that some specific shortcut strategies produce correct results. For instance, in the min strategy, calculation of, for instance, the problem 2 + 5, can be facilitated by counting on from the larger addend (the 5), as when solving by counting on from 2 [22–24]. Note that according to Baroody and Gannon [25] only those children have completely understood the concept of commutativity who comprehend addition as a binary rather than as a unary operation. The binary view of the addition of two numbers would, for instance, interpret 2 + 4 as summing two independent cardinalities, 2 and 4. The unary view would interpret 2 + 4 as the addition of 4 more units to 2. In this case the two addends play an asymmetric role, one is added to the other, rather than that they are added *together*. The assertion that, for instance, the sum of two and four more is equal to four and two more still involves a unary interpretation of addition. Weaver [26] calls such an assertion "pseudocommutativity" as it does not describe the property of an operation. Though such statements as 2 + 4= 6 and 4+ 2 = 6 are mathematically equivalent, psychologically they imply different meanings – even for adults.

It is debatable whether using the min-strategy implies that a child understands the commutativity principle. Geary [27] pointed out that it is not clear when children “explicitly understand commutativity as a formal arithmetical principle” (p. 791). The difficulties in answering this question are due to the fact that researchers by no means agree upon the characteristics of procedural or conceptual knowledge that must be given in order to conclude that children have an explicit understanding of a mathematical concept or how to best assess this knowledge [28,29]. For example, researchers assess the understanding of mathematical concepts by examining accuracy and solution latencies on diagnostic problems, analyze children’s eye movements, or have children judge whether or not a given strategy is admissible [30–35]. Mostly, these methods are combined with interviewing the children after performing the calculation, to clarify how the problem was solved. For example in the study by Canobi et al. [36] children had to solve commutative problems. After each problem, the interviewer asked the children how they “worked out the answer”, and prompted them when necessary. For instance, children who counted were asked, “What was the first number you said as you started counting?” Children were assumed to have used their conceptual knowledge of commutativity if they reported solving a problem by referring to a related, immediately preceding problem, for instance, "I saw that 2 + 7 had the same numbers as 7 + 2 (the preceding problem), so I knew the answer to 2 + 7 was 9 as well." It is unclear whether asking children to explain their solution strategies triggers the use of the strategies during the investigation. In some cases, children were even asked explicitly to use efficient strategies [37]. It is conceivable, that children look at the problems more attentively when they are asked to verbalize their strategies. Consequently, the question whether the commutativity knowledge that a child possesses even allows for spontaneous recognition and usage of shortcut options remains open.

The principle of commutativity allows for two different types of efficient strategies exploiting the order-irrelevance principle. For some mental arithmetic problems, addends *within* a problem can be rearranged in order to simplify it. The *ten-strategy* consists of reordering multiple addends within a problem in order to exploit the circumstance that (non-neighboring) numbers add up to ten. The solution of a problem like 4+8+6 can be simplified by transforming the task into (4+6) +8. For other mental arithmetic problems, however, computation itself can become superfluous if one recognizes that the same addends had been presented (though in different order) in the previously solved problem. This requires the application of the concept of commutativity *between* problems. In the *addends-compare strategy*, effort is spared based on (a) the identification of the fact that two problems consist of identical addends in a different order and (b) the knowledge that solving the first problem makes the calculation of the second one unnecessary (e.g., 3+8+5= after 5+8+3=). Either shortcut speeds up the solution process relative to problems that do not allow for the shortcut.

Note that some researchers use the term *associativity* instead of *commutativity* when an addition or multiplication problem has more than two addends or factors [38]. Other researchers [36] refer to c*ommutativity* as the property that problems containing the same terms in a different order have the same solution (independent of the amount of terms), whereas *associativity* is the property that problems in which terms are decomposed, and recombined in different ways, have the same answer [*(a + b*)* + c = a + (b + c*)]. In line with the definition provided by WIKIPEDIA [https://en.wikipedia.org/wiki/Associative_property, retrieved May, 20^th^, 2013] we use the term commutativity for our arithmetic problems as they involve changes in the order of operands in the equation. Associativity refers to the issue that in an expression with two or more subsequent occurrences of the same associative operator, the order in which the operations are performed does not matter as long as the sequence of the operands is not changed. Rearranging the parentheses will not change the value of the expression, e.g. (5+2) +1=5+(2+1)=8. Yet, in commutativity, the operands commute – they change in order. Commutativity justifies changing the order or sequence of the operands within an expression while associativity does not. (5+2) +1=(2+5) +1 is referred to as an example of commutativity, but not of associativity, because the operand sequence changed when the 2 and 5 switched places.

The current work explored the quality of knowledge about the commutativity-principle by testing the spontaneous application of different shortcuts based on the principle (a) with a paper-and-pencil approach in the classroom as well as with (b) computerized testing involving eyetracking. In the classroom setting, children from different grades (and university students) worked on different booklets with addition problems. All participants received booklets that allowed for the addends-compare strategy or for the ten-strategy. In addition, baseline booklets were presented that lacked the opportunity of using any shortcut strategy but otherwise were matched in structure and difficulty of the problems. Children should solve more addition problems per time on the shortcut-based booklets as compared to the control booklets. We expected that the interrelation between the addends-compare strategy use and the ten-strategy use would increases with age. In a follow-up we aimed at more detailed process data on spotting and spontaneously applying the commutativity-based shortcut options. We tested an independent sample of primary school children individually recording solution times problem-by-problem as well as eyetracking parameters indicative of the shortcuts. As the eyetracking study was developed based on the material and procedure of the paper-and-pencil study, we will describe the former in detail and later point out the adaptations necessary for the eyetracking variant.

## Method

### Participants

A total of 364 elementary school children and 164 university students participated in the paper-and-pencil-based study and 26 in the eyetracking study (Table 1 for descriptive statistics). Children were recruited from three different elementary schools of Berlin. The research procedures described below were approved in a peer review process for applying for public funding of the research (German Research Foundation, DFG) and were completed in accordance with approval from the Institutional Review Board of the Department of Psychology at Humboldt-Universität, Berlin. We ensured written informed consent of the parents in collaboration with the schools. University students were enrolled at Humboldt-Universität Berlin and received course credit. Either group was provided with advance information concerning the content of the study (calculate mental arithmetic problems) and was informed that participation was voluntary. Participants were also informed that data analysis would not entail charting person-specific results (i.e., names were not collected with the data).

**Table 1:** Sample data and time provided per booklet in the paper-and-pencil-based study.

Grade	N (females)	Mean Age (SD)	Seconds for addends-compare / ten-strategy booklets
2	124 (72)	7.5 (.3)	240/120
3	76 (42)	8.7 (.5)	150/45
4	120 (54);*46	9.7 (.6)	120/45 (*240)
7	*44 (21)	12.9 (.6)	180*/40
University	164 (99);*92	25.5 (7.7)	60/25 (*120)
Eyetracking study	26 (12)	9.1 (2.3)	Participant-paced

* difficult addends-compare tasks

### Materials

Booklets providing the opportunity to use the addends-compare strategy (addends-compare booklets) and booklets allowing for the ten-strategy (ten-strategy booklets) were both accompanied by baseline booklets lacking such shortcut options (Table 2). All participants first worked on the addends-compare booklet (or the respective baseline booklet – with the order being counterbalanced to control the impact of warm-up effects) and were then transferred to the ten-strategy booklet followed by its baseline booklet. Booklets with shortcut options and matched baseline booklets consisted of arithmetic problems of the same size. In order to minimize the opportunity for copying from the neighbour in class-based assessment we used two parallel versions A and B of each booklet.

**Table 2:** Examples of the first six problems of each problem type (*addends-compare* and *baseline* *problems*) in the parallel sets A and B.

Set A		Set B	
Addends-compare	Baseline	Addends-compare	Baseline
*Small addends*			
3+5+4=12	5+3+4=12	4+3+5=12	4+5+3=12
4+9+8=21	8+9+4=21	5+7+9=21	8+4+9=21
4+8+9=21	6+7+8=21	5+9+7=21	7+8+6=21
6+2+5=13	5+2+6=13	2+6+5=13	2+5+6=13
9+7+2=18	2+7+9=18	4+5+9=18	7+9+2=18
2+9+7=18	9+4+5=18	5+4+9=18	5+9+4=18
*Large addends*			
5+17+46=68	6+17+45=68	36+5+27=68	37+5+26=68
44+48+3=95	24+68+3=95	3+34+58=95	6+27+62=95
44+3+48=95	36+7+52=95	3+58+34=95	4+53+38=95
59+2+24=85	49+4+32=85	39+4+42=85	24+9+52=85
35+34+8=77	36+33+8=77	43+28+6=77	48+25+4=77
8+35+34=77	8+45+24=77	28+43+6=77	23+46+8=77

The results printed in italics had to be filled in by the participants.

Addend-compare booklets (and the accompanying baseline-booklet) contained 30 different addition problems (Table 2). Each consisted of three different addends between 2 and 9 (maximum result was 24; 0 and 1 were excluded as addends). The different problems yielded the same sums for version A and B. Within a problem, each addend occurred only once. To control the use of the min-strategy, the position of small and large addends within the problems was balanced across the different problems. The addends-compare strategy could be applied on two of the six addition problems on each of the five pages of the addends-compare booklets (i.e., same addends as in prior problem, but different order).

In order to explore the impact of task difficulty on the spontaneous application of commutativity-based shortcuts, we increased calculation difficulty of the addends-compare booklets (and the respective baseline booklet) for some of the participants (Tables 1 and 2). We administered the difficult variant to 46 of the fourth graders (74 with the simpler version) and 92 of the university students (72 with the simpler version). All seventh graders worked on the difficult version as pilot-testing had suggested that in higher grades the simple math-problems could lead to motivational problems for some of the students.

Ten-strategy booklets and the respective baseline-booklets were composed by arranging three-addends arithmetic problems such that the first and the last addend add up to ten (maximum total was 19; 0 and 1 never occurred as addend; ties only occurred for the number 5, Table 3). The 20 problems were distributed over two pages. We did not vary addends size in the ten-strategy booklet and the respective baseline booklet, but did so for the addends-compare booklets provided before (see above). This allowed us to compare potential transfer effects between commutativity-based strategies either from large to small addends vs. from small to small addends. If activation of commutativity knowledge generalizes beyond the set of specific problems, ten-strategy usage should be similar no matter whether the tasks provided in the booklets before (addends-compare and baseline booklet) were both small vs. first large and then small (cf. [8], for a review of the issue of abstractness in transfer).

**Table 3:** Examples of the first five problems of each problem type (*ten-strategy* *problems* and *baseline*) in the parallel sets A and B.

*Set A*		*Set B*	
*Ten-strategy*	*Baseline*	*Ten-strategy*	*Baseline*
4+5+6=15	4+3+8=15	6+5+4=15	8+4+3=15
3+2+7=12	3+5+4=12	7+2+3=12	3+4+5=12
5+6+5=16	8+5+3=16	5+9+5=19	8+3+5=16
7+4+3=14	2+5+7=14	3+4+7=14	5+2+7=14
2+7+8=17	9+3+5=17	8+7+2=17	5+3+9=17

For ten-strategy problems two non-neighbouring addends added up to ten. The results printed in italics had to be filled in by the participants.

### Procedure

Students were tested in groups of up to 25 participants (university students up to 55) in a classroom situation. One experimenter instructed the whole class. Students were told to work on the problems on each page of the booklet from top to bottom and not to skip any problem. When the experimenter said “start”, participants should begin with their calculations and upon the experimenter saying “stop” they should immediately lay down their pencils. To ensure that the instructions were followed, three to five additional experimenters observed small groups of participants in the classroom. The experiment started with a practice phase in order to familiarize participants with the task requirements, especially the start and stop rules. They were given two minutes to solve six practice problems.

Students were instructed to solve the problems as quickly and as correctly as possible. Additionally, they were informed that it would be nearly impossible to solve all problems during the period of time given for each booklet. The period of time given to solve the problems in each booklet differed depending on task type and grade (Table 1). The experimenter measured the time with a stopwatch.

### Procedures Specific for the Eyetracking Study

Participants were tested individually with a 250Hz video-based eyetracker (SMI RED250) sitting at approximately 50cm distance from a 22″ TFT monitor. After a five-point calibration and a single example problem, we presented six problems in black on grey background simultaneously on the screen. Digits were approximately .5 cm wide and 1 cm tall. The distance both between the lines and columns of digits was 5 cm. Starting with the first of six arithmetic problems on a page, the experimenter located the cursor right to the equal sign. When the participant uttered an answer to the problem, the experimenter immediately typed it in so that it was displayed on the screen. Solution time was recorded based on the first key-press. When the experimenter afterwards placed the cursor right of the equal sign of the next arithmetic problem on the page, the answers given to prior problems on the page remained visible. Thus participants could re-examine the previous problems and answer in order to check whether the addends-compare strategy would apply.

Material was identical to the paper-and-pencil study (small addends variant) with few exceptions. We used the first two pages of each booklet. Furthermore, we changed the order of booklets such that we could obtain a detailed measure of the addends-compare strategy. Participants first worked on addends-compare and baseline booklets so that we could obtain problem-by-problem solution times and fixation patterns related to spontaneous usage of the addends-compare strategy. Next we obtained data to judge how large the effect of spontaneous usage was by comparing it to instructed usage. For this we reminded participants of the commutativity principle and the addends-compare strategy. Afterwards, participants worked on an addends-compare booklet followed by a baseline booklet. Finally, they worked on a ten-strategy booklet followed by the respective baseline. Note that half of the participants started with working on an addends-compare booklet, followed by a baseline booklet and another addends-compare booklet. The other participants started with baseline before proceeding to and addends-compare booklet and yet another baseline booklet. We included this variation in order to explore whether it is helpful for spotting and using a shortcut option to present it in the material already at the very beginning of the experiment. As there was no clear indication that this was the case, we collapsed across these two variants.

## Results

### Paper-and-Pencil

We will first present the results of the paper-and-pencil study and then the eyetracking results. Mean processing time was calculated separately for each participant by dividing the individual amount of processed problems in each booklet by the total time given for the entire booklet in the respective grade group. Participants who completed less than 3 problems in one of the 4 booklets or solved more than 50% of the problems incorrectly were excluded from further analyses. These criteria led to the exclusion of four second graders and one third grader. All statistical tests were performed with an alpha level of .05.

#### Addends-compare strategy

**Figure 1:**
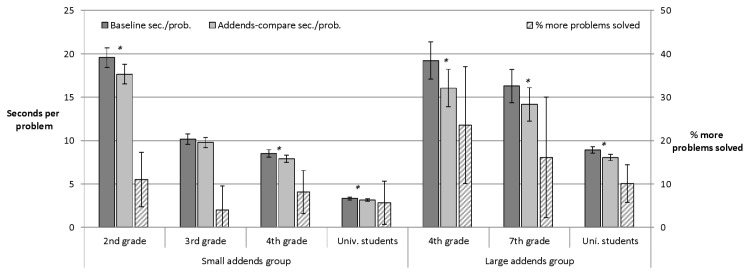
Addends-compare strategy. The mean processing times in seconds per problem for booklets allowing for a shortcut with the addends-compare strategy (light grey) and the matched baseline booklets (dark grey). The error bars contain the 95% within-participants confidence interval for the comparison between the two booklets. Asterisks indicate significant comparisons. Bars with dashed lines display the relative benefit on addends-compare booklets relative to baseline booklets. The error bar displays the 95% confidence interval of the comparison with zero benefit.

Figure 1 depicts the mean processing times in seconds per problem for addends-compare (light grey) and matched baseline booklets (dark grey). Processing times were calculated individually based on the overall number of solved problems per booklet. We included incorrectly solved problems in order to estimate the benefit by addends-compare or ten-strategy booklets in a conservative way. For instance, participants should be faster (and not just less error prone) when applying the simplifying ten-strategy. The analyses were separated for children of different grades and for those working on small vs. those working on large addends. Mean error rates are displayed in Table S1.

Average calculation time per task was significantly shorter for addends-compare booklets than for baseline booklets for the various subsamples of different grades tested with either small or large addends (Figure 1 and Table S1). Third graders formed the only exception. For them the calculation time on addends-compare booklets was not significantly shorter than the calculation time on baseline booklets. The subsample of third graders also deviated in the second measure, the error rates. It was the only subgroup with a significant difference in error rate between the booklets, *t*(74) = 2.77, *p* = .007, with a higher error rate on the addends-compare as compared to the baseline booklets. Note that we used t-Tests for the within subjects comparisons between booklets with vs. without option for simplification of task processing. All results were confirmed by Wilcoxon Signed Ranks Test – non-normality of difference scores was not invalidating the results. In most conditions we found (as expected) a benefit in solution time for booklets allowing for a shortcut (rather than single significant results amongst several null-effects). Therefore we did not apply Bonferroni correction.

As one would expect, students in higher grades were generally faster than students in lower grades. Also subsamples tested with large addends needed more time per task than subsamples tested with small addends. Interestingly, the baseline task seemed to be equally difficult for fourth graders tested with large addends and for first graders tested with small addends. Yet, the increase in speed on the addends-compare booklet relative to the baseline booklet was larger for the fourth graders than for the second graders.

In additional analyses we wanted to compare the commutativity benefit on easy vs. difficult booklets in the same grade. Thus, differences in performance on baseline-booklets had to be controlled for. We calculated a relative benefit measure which is an index for the extent to which a participant benefited from addends-compare booklet relative to baseline booklet. For participants who solved more tasks on the addends-compare booklet as compared to the baseline booklet, we divided the corresponding difference score by the number of tasks solved on the baseline booklet. If for instance, a participant solved 12 problems on the addends-compare booklet and 10 on the baseline booklet, this person had a 20% benefit [20% =100 * (12-10)/10]. For the case that a person solved more problems on the baseline booklet than on the addends-compare booklet, we calculated a negative relative benefit by dividing the difference by the number of problems solved on the addends-compare booklet. If for instance a person solved 11 problems on the baseline booklet and 10 on the addends-compare booklet, than this person would have been assigned a benefit of -10% [-10% = 100 * (10-11)/10]. We used this relative measure because it provides symmetric estimates so that the average relative benefit over participants will be zero in case the average number of problems solved on addends-compare and on baseline booklets is identical. Note that even in the case of identical average numbers of problems solved in either booklet, one would get an artifactual positive average benefit if one uses the number of problems solved on the baseline booklet as divisor irrespective of the direction of the difference. This is not the case, if one changes the divisor depending on whether the difference is positive or negative. We briefly illustrate this issue. Above we used the example of a participant who solved 12 problems on the addends-compare booklet and 10 on the baseline booklet. This person had a 20% benefit [20% =100 * (12-10)/10]. If we’d also divide by the number of problems solved on the baseline booklet for a person who solved 12 problems on the baseline booklet and 10 problems on the addends-compare booklet, this would not lead to a symmetrical result, but to -16,67% = 100* (10-12)/12. The average of such a relative measure (i.e., averaging across 20% and -16.67%) would thus be positive, even if the average number of problems solved is identical for either booklet (i.e. on average 11 problems solved for either booklet).

Furthermore one could suspect that a relative measure artificially increases the estimate of benefit for participants starting very low. In the Supporting Information (Text S1 and Figure S1) we show that this does not seem to apply here. Rather we found that large gains on booklets with shortcut option were in tendency more pronounced for students with low baseline performance for both, the relative as well as the absolute benefit measure.

As suggested by Figure 1, fourth graders profited more from addends-compare booklets relative to baseline booklets if large addends were used rather than small addends, *t*(118) = 2.52, *p* = .013. For university students, the difference between the variant with large vs. small addends was not significant, *t*(162) = 1.34, *p* = .181.

In summary, we observed that students of all age groups spontaneously applied the addends-compare shortcut. Different from research using more direct cues and questions to tap into knowledge of a mathematical principle, rate of spontaneous usage was substantial but modest. Even for adult students there was no ceiling effect, more calculation time could have been saved by applying commutativity knowledge more thoroughly.

#### Ten-strategy

**Figure 2:**
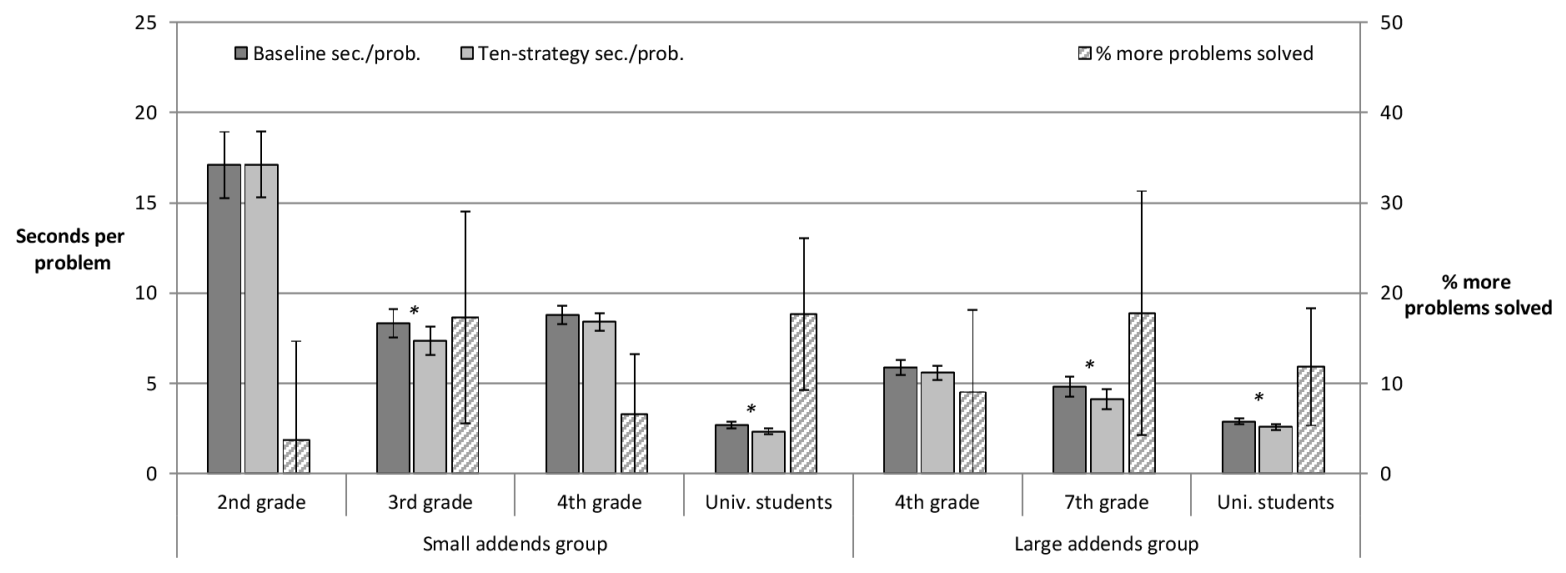
Ten-strategy. Measures analogous to the ones in Figure 1 are displayed for the ten-strategy.

From grade three onwards, participants took less seconds per arithmetic problem on booklets allowing for the ten-strategy as compared to the baseline booklets. As detailed in Figure 2 and Table S1, this difference was significant for third graders, seventh graders and university students. For fourth graders the benefit on sheets with shortcut option was not significant. The pattern was highly similar for the relative benefit of ten-strategy booklets over baseline booklets. It was significant for third graders, seventh graders and university students. For either subgroup of fourth graders the relative benefit was at the border of significance, *p* = .051 and *p* = .05 for small and large addends respectively. Furthermore, fourth graders and university students had a significantly lower error rate on ten-strategy booklets as compared to baseline booklets.

As the ten-strategy booklet was provided after the addends-compare booklet, there could be transfer from using one commutativity-based shortcut on the former to applying another commutativity-based shortcut on the latter. On the one hand, one could hypothesize that participants who work on addition problems with small addends in all booklets have an advantage in applying the ten-strategy after having applied the addends-compare strategy first. Usage of associations linking one strategy to the other one might be more likely if the context is highly similar (i.e., problems with same range of addends). On the other hand, difficult problems might lead to stronger usage of the addends-compare strategy, which in turn might provide a stronger basis for transfer – even when taking into account that the material allowing for one or the other shortcut is less similar. In order to explore these options we tested whether there is an effect of addends size in the addends-compare problems on performance on the ten-strategy booklets. There was no evidence for that transfer from addends-compare to the ten-strategy might suffer from a change in addends size. An analysis of variance on the relative benefit of ten-strategy booklets relative to the baseline for fourth graders and university students, yielded no effect of addends size, *F* < 1, nor an interaction of grade and addends size on addends-compare booklets, *F* = 1.14. There was a tendency towards a main effect of grade, as the relative benefit was larger for the university students as compared to the fourth graders, *F*(1, 280) = 3.18, *p* = .076.

#### Correlations between addends-compare and ten-strategy

In the next step we analysed for each subsample the correlation between (a) addends-compare strategy and (b) ten-strategy. For each participant, we (a) calculated the difference between the number of problems solved in addends-compare booklets and the number of problems solved in baseline booklets, as well as (b) the difference between the number of problems solved on ten-strategy booklets and the respective baseline. We preferred the simple difference measure over the measure of relative benefit as it reduced the impact of outliers. In addition, we compared the Pearson correlation coefficients reported below with rank order correlations (and found no differences in pattern).

As detailed in Table 4, we obtained positive correlations between the two indicators of commutativity-based strategies from third grade onwards. Unexpectedly, there was no correlation for fourth graders working on addends-compare booklets with small addends (while there was for fourth graders with the version with large addends). Exploring this unexpected effect, we split the subsample of fourth graders (small addends) for participants with high vs. low baseline performance. To do so, we took the average performance of the two baseline booklets and separated the two subgroups of participants by a median split. For students with a low calculation competencies, the correlation was negative, *r*(38) = -.497, *p* = .002. Participants who benefitted from the addends-compare booklet were unlikely to benefit from the ten-strategy booklet, and vice versa. For the group of students with calculation competencies, there was a positive correlation, *r* = (36) = .438, *p* = .008. Participants who profited from addends-compare booklets also tended to profit from ten-strategy booklets. Thus, the lack of a correlation in the fourth graders working on small addends problems resulted from two subgroups within the fourth graders which showed reverse correlations. There was no other condition with a significant difference in correlations of participants with high vs. low baseline (Table 4).

**Table 4:** Correlations between (a) addends-compare strategy and (b) ten-strategy.

		*r* whole group	*p*	*r* low baseline	*r* high baseline	*p* of diff. high vs. low
Small addends	2nd grade, n=120	-.031	.74	-.113	-.028	.324
	3rd grade, n=75	.281	.015	.159	.365	.178
	4th grade, n=74	-.003	.979	-.497	.438	<.001
	Univ. students, n=72	.238	.045	.258	.068	.787
Large addends	4th grade, n=46	.366	.012	.492	.231	.831
	7th grade, n=43	.37	.015	.308	.247	.581
	Univ. students, n=92	.119	.257	.029	.083	.401

Again we obtained no hint that transfer from the addends-compare strategy to the ten-strategy would diminish if different sizes of addends were used. To the contrary, due to the (unexpected) lack of a correlation in the fourth grader sample working on small addends, the correlation between the strategy indicators was significantly larger, *Z* = -2.0, *p* = .023, if problems were dissimilar (large addends in addends-compare booklets) rather than similar (small addends in either booklet). Of course we are cautious to interpret this finding beyond the statement that there was no sign of reduced transfer from large to small addends as compared to from small to small addends.

### Trial-by-Trial Solution Times and Eye Fixation Data

**Figure 3:**
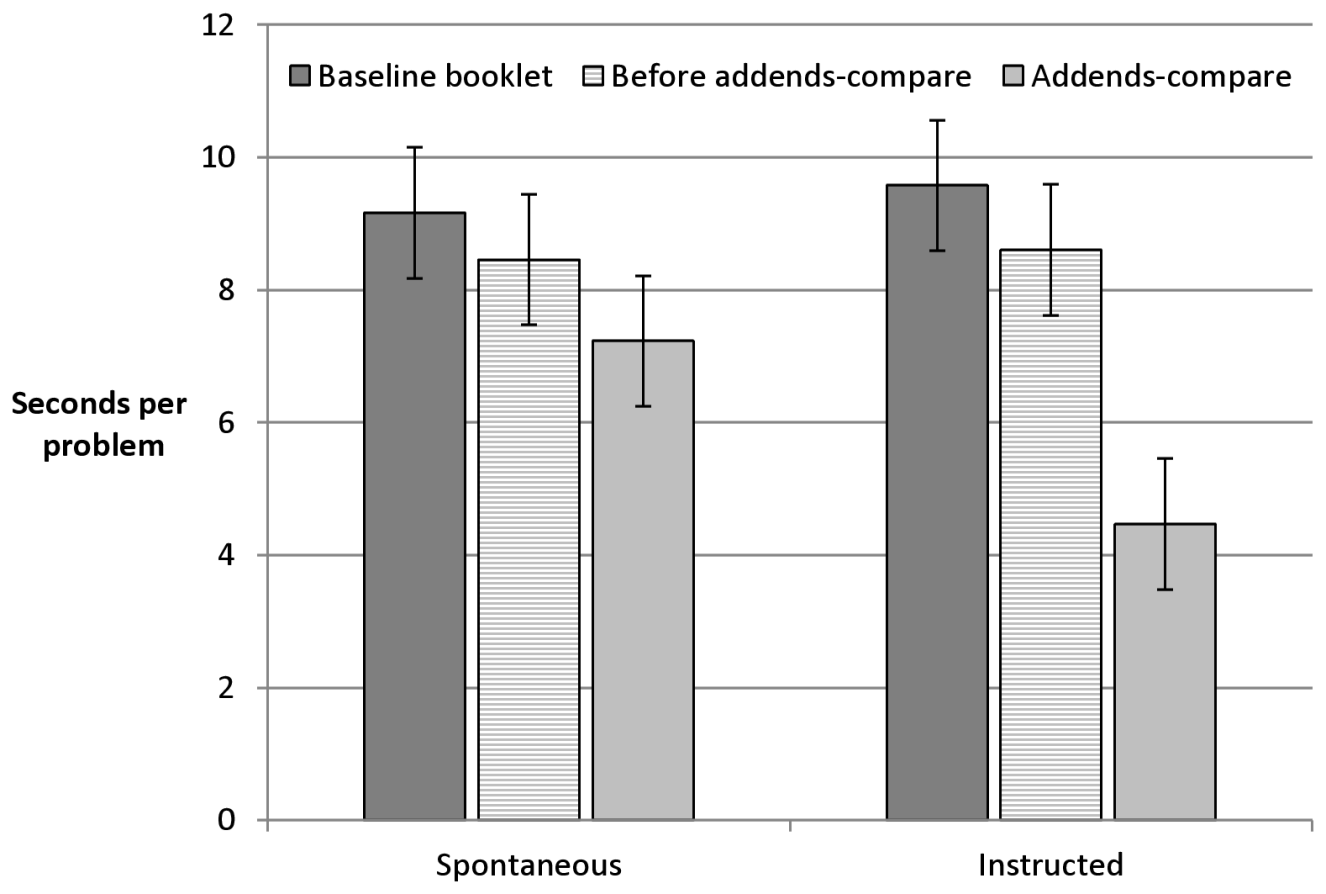
Addends-compare strategy on the level of single problems. Calculation time per problem type in the eyetracking study. Error bars indicate the 95% within subjects CI according to Masson & Loftus [53] based on the error term of the interaction.

The follow-up study provided solution time data on the level of single arithmetic problems as well as eyetracking data indicating the extent to which participants were referring back to previous problems or checking subsequent problems when solving the current equation. With respect to the addends-compare strategy, we first analysed solution times for problems in three different types of contexts: (1) problems on baseline booklets, (2) problems preceding addends-compare problems and (3) addends-compare problems. The latter two consisted of identical addends (in different order). Thus, we compared problems displayed on the same page that allowed vs. did not allow for the addends-compare strategy and contrasted this detailed analysis with a comparison involving baseline booklets. The detected benefit for addends-compare booklets can in principle be a consequence of faster solutions of the problems within this booklet. However, it can also result from costs when solving the baseline booklets: Participants might lose time on baseline booklets when searching for options to apply the addends-compare strategy (which do not exist). Figure 3 suggests that both factors might have played a role. When strategy application was spontaneous, problems on baseline booklets were solved slowest and addends-compare problems were fastest, while solution time for problems preceding the addends-compare problems lay in between. When participants were explicitly instructed to apply the addends-compare strategy, the solution time benefit brought about by addends-compare problems was by far larger than potential search-related costs on baseline booklets. An ANOVA with the within-subjects factors problem type (baseline, preceding addends-compare, addends-compare) and instruction (spontaneous vs. instructed) confirmed a main effect of problem type, *F* (1.89, 47.32) = 20.28, *p* < .001, and an interaction of instruction and problem type, *F* (1.42, 35.52) = 8.47, *p* = .001 (both Greenhouse-Geisser corrected; no main effect of instruction, *F* = 1.2). Single comparisons indicated that the potential search costs on baseline booklets (compared to problems preceding addends-compare problems) were not significant (ps > .21). Error rates indicated a floor effect for many of the participants and no robust differences between the conditions (Table S2).

**Figure 4:**
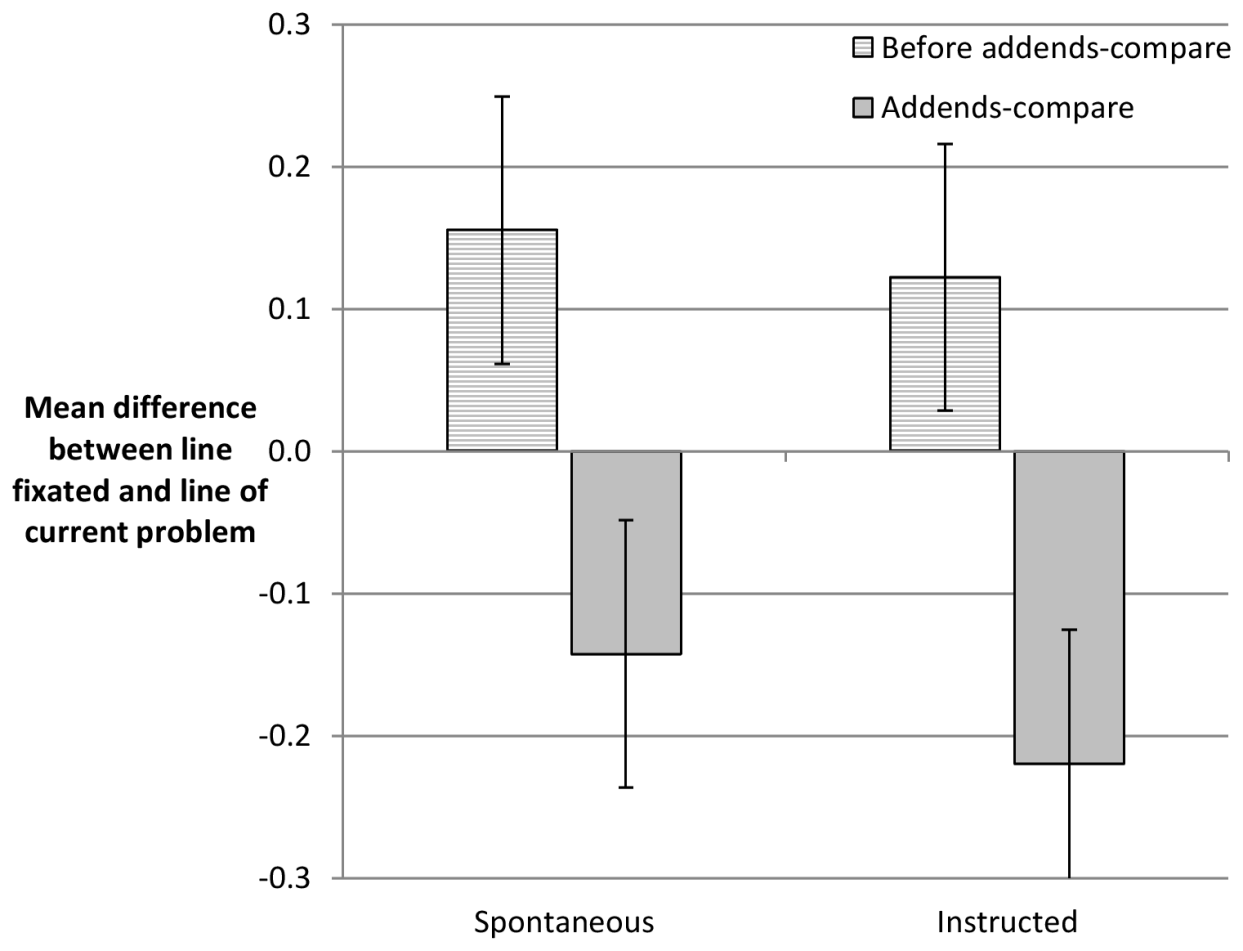
Eyetracking indicator of addends-compare strategy. Mean difference between current line of current problem vs. current line fixated. Negative values indicate fixations on preceding problems while positive values result from fixations on subsequent problems. Like in Figure 3, error bars indicate the 95% within subjects CI.

Next we explored how fixations reflected the processing of the addends-compare problems. For this, fixations were determined in the eyetracking data as clusters of similar subsequent screen coordinates – as indicated by an accelerations below 40°/s (otherwise a saccade). As participants had to eventually fixate all the columns and lines with addends, we could use the distribution of fixations in the matrix off addition problems to verify the boundaries between addends. Based on the distribution of fixations we determined, at which addend of which arithmetic problem a specific fixation was directed. As participants were working from top to bottom on pages with six arithmetic problems, we determined for each fixation whether it was located in the line of the current problem (*M* = 52.3% of the fixations) or on lines of previous vs. subsequent problems. Figure 4 shows the mean difference score of the line fixated vs. the line of the current problem. We computed this difference score separately for the addends-compare problems as well as the problems preceding them. It is evident that fixations off the line of the current problem were placed in accordance with affordances of the addends-compare problems and the addends-compare strategy. When working on the problem prior to the addends-compare problem, participants were in tendency placing their off-the-line fixations ahead, which would be consistent with checking in advance the next problem. However, when working on the addends-compare problem, participants were rather fixating previous problems. This would be in line with the idea that participants check previous problems for applicability of the addends-compare strategy. An ANOVA with the within-subjects factors problem type (preceding addends-compare, addends-compare) and instruction (spontaneous vs. instructed), confirmed that problem type lead to an effect on the line fixated, *F*(1, 25) = 60.48, *p* < .001. There was neither a main effect of instruction nor an interaction with problem type (*F*s < 1).

Solution times for ten-strategy booklets were faster (*M* = 7.1s) as compared to baseline-booklets: *M* = 8.2s, *t*(23) = 2.49, *p* = .021. Data of three participants were lost due to technical problems. The correlation between the ten-strategy benefit and the addends-compare benefit just missed significance *r*(23) = .4, *p* = .058. There was no robust correlation of either benefit with age (both rs = -.25, ps > .222).

**Figure 5:**
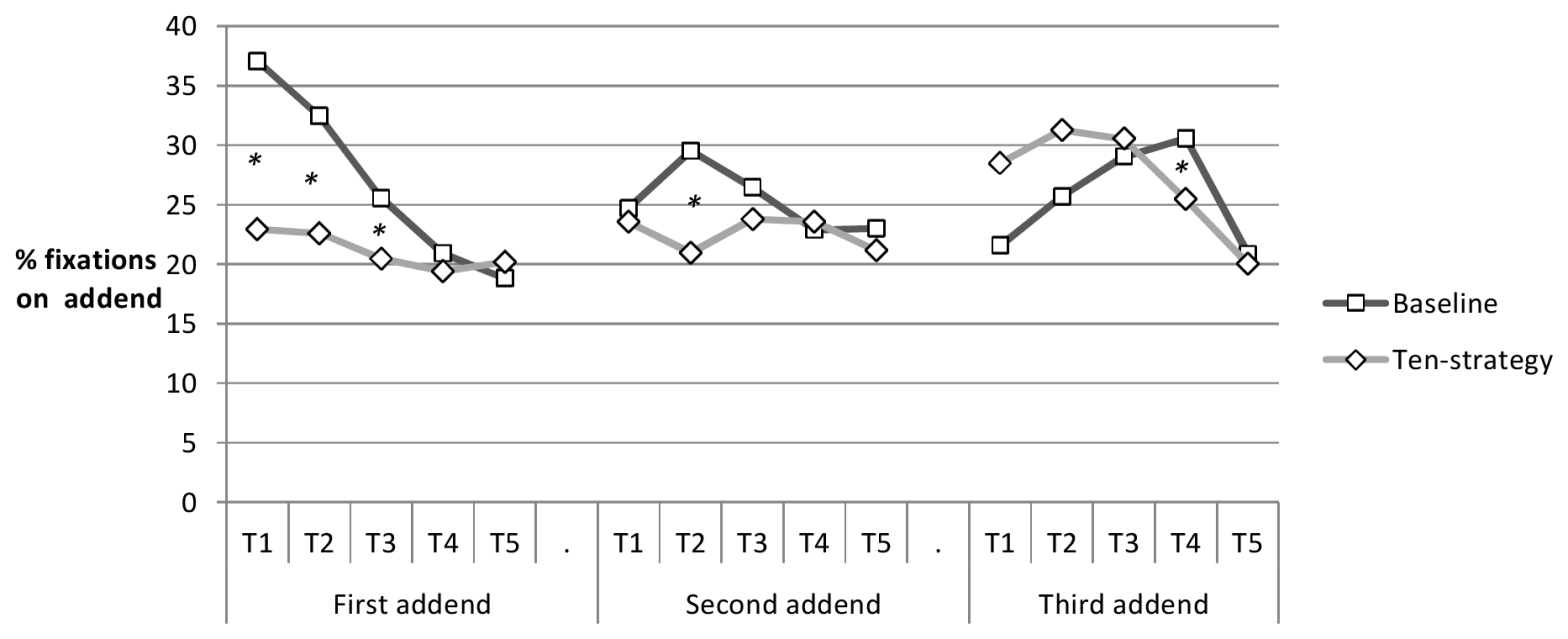
Eyetracking indicator of ten-strategy. Fixation frequency on first, second, and third addend on ten-strategy and baseline booklets charted for the time-course of the problem-solving episodes. For instance, the graph indicates that more than 35% of the fixations taking place within the first fifth of the time a participant has worked on a problem on a baseline booklet were located at the first digit.

Fixation patterns reflecting the ten-strategy are depicted in Figure 5. For each fixation we determined whether it was closest to the first, second, or third addend. We charted the corresponding frequencies for the ten-strategy vs. baseline booklet ordering fixations according to time quintile (i.e., first 20%, second 20%... …fifth 20% of fixations while solving one problem). For the baseline booklet many of the first 20% of the fixations while solving a problem were located on the first addend. Many of the fixations of the second time-quintile were located on the second addend. The third addend was fixated to an increasing amount the longer a participant had worked on the current problem. Thus, for the baseline-booklet fixations showed a pattern ordered in time and space with first fixations rather falling on the first addend and later fixations on the second or third addend. For the ten-strategy booklet, however, fixations did not follow this order. In line with the ten-strategy, participants checked all addends early on, favouring the first and third addend over the second one in early fixations. Such a pattern can be expected when participants first add the outer addends (which add to ten) before taking care of the addend in the second position. Keeping in mind the interdependency of the frequency data and avoiding capitalization on chance we tested differences in the two distributions averaged across participants, X^2^(15) = 37.52; *p* < .0001.

## Discussion

Knowledge of mathematical principles is especially helpful if we recognize without instruction or direct cues when we can apply it for efficient processing of arithmetic problems [4]. As a basis for research detailing when and how spontaneous application of arithmetic shortcuts takes place and how it is related to conceptual knowledge of mathematical principles, we introduced two variants to unobtrusively test for spontaneous shortcut application. With the paper-and-pencil-based measure, we explored the development of the spontaneous use of two strategies derived from the mathematical concept of commutativity in primary school. Employing computerized testing and eyetracking, we identified fixation patterns related to either of the two commutativity-based shortcuts. In line with the addends-compare strategy, participants were fixating previous problems when currently working on a problem containing the same addends in different order as the previous one. On other problems, they seemed to check subsequent problems in advance for whether or not the addends-compare strategy could be applied. Material allowing for a shortcut based on the ten-strategy led to early fixations on first and third addend – as would be beneficial when exploiting the fact that these addends add up to ten.

The commutativity-based addends-compare shortcut was spontaneously used from second grade onwards. This is in line with studies showing basic understanding of the concept of commutativity before entering school [16–18,20] and – with respect to adding numbers – in first graders [16,21]. The ten-strategy, however, was spontaneously applied only after second grade. Spontaneous identification of information relevant to solve a problem efficiently has been discussed as a marker of expertise consisting of integrated procedural and conceptual knowledge [5–10]. In line with this, our findings suggest that commutativity knowledge was to some extent abstract [8]. On the one hand, we obtained spontaneous discovery and application of shortcut options with task material which was infrequently used in teaching (three-addends problems, cf. [37]) and not previously presented in the context of teaching commutativity-based shortcuts. On the other hand, comparison between children who worked on addends-compare booklets with small vs. large addends provided no indication for that similarity of task material influenced the amount of transfer between the addends-compare and the ten-strategy. There was no hint towards increased transfer between the addends-compare and the ten-strategy if addends were similar (i.e., of the same size) in both booklets rather than of different size. Though awaiting replication (i.e., fully crossing the possible different orders of ten-strategy and addends-compare booklet and addends size), transfer seemed to be stronger from large to small addends as compared to transfer from small to small addends.

From third grade onwards, there was a correlation between the usage of either commutativity-based shortcut. Like the above findings, this is consistent with the development of integrated knowledge which includes conceptual knowledge about the commutativity principle as well as procedural knowledge for different commutativity-based strategies. However, future work should include tests of spontaneous shortcut application together with direct tests of conceptual knowledge in order to provide a stronger basis to evaluate this claim. Furthermore, by focusing on strategy use we do not imply that different addition strategies develop first while conceptual understanding of commutativity develops only later. We set aside the question whether concepts or strategies set the starting point in the development of mathematical concepts. Common to most current views [13,39–41] is the assumption is that procedural and conceptual knowledge develop *iteratively* with small increases in one leading to small increases in the other which in turn trigger new increases in the first. The endpoint of development should, in the best case scenario, be marked by an integrated concept. We assume that this concept should be reflected in the spontaneous use of adaptive strategies.

According to the course material and explanations from the teachers, basic knowledge about the commutativity principle had been taught by second grade. Specifically, children had been instructed concerning the procedural implications of the principle for two-addends tasks and had practiced them in a blocked manner. As teaching was not exhaustively covering the issue, one would not expect all students to spontaneously apply commutativity-based shortcuts. We observed that the students who showed high abilities on the baseline booklets did not profit the most from the shortcut options (see Text S1 and Figure S1 for detailed analyses). Rather, absolute and relative benefits were larger for students with lower performance on the baseline booklets. It is thus not likely that correlations between the indicators of the two shortcut strategies are driven by general mathematical ability or general intelligence. Rather differences in procedural and/or conceptual knowledge as well as differences in the motivation to apply shortcuts might account for the interindividual variability. For instance, for participants with low baseline performance the costs of switching the mode of task processing between (a) calculation and (b) checking for shortcut options might be small compared to the efforts in solving the problems with calculation [42]. However this issue remains open for now. Higher general intelligence has been reported to be linked to smaller costs in keeping multiple ways of task processing simultaneously active as well as to smaller costs of switching between different ways of task processing [43]. A developmental study with young chess players suggests that a high amount of practice rather than high intelligence is the most important prerequisite of developing the capability to recognize visual patterns linked to efficient strategies [44].

We observed that spontaneous usage of the addends-compare strategy was less pronounced than instructed usage. While there was a general improvement in calculation speed with grade, there was no ceiling effect in spontaneous shortcut usage [45]. Even for the university students, there was ample room to further increase task efficiency by exploiting the order irrelevance principle. At first, this seems surprising given that Baroody et al. [46] showed that commutativity-based computational strategies were applied by more than 80% of second and third graders. However, our approach differed in several regards. We used relatively unfamiliar three-addends problems and focused on spontaneous strategy usage in a testing setting that did by no means highlight that we were specifically interested in *how* students solved the tasks. Rather, the class based testing placed emphasis on the number of tasks solved per unit of time. Individualized testing and questions concerning the paths to the solution of a problem might trigger a search for more efficient modes of solving the problems and by this help students to find and apply shortcut options. For instance, Robinson and Dubé provided evidence that spontaneous strategy application was lower than when verbal reports were required [47–49].

The effect of addends size and the correlation between shortcuts based on the same principle might hint at a potential means to increase the spontaneous usage of a commutativity-based shortcut and by this foster transfer to a different commutativity-based strategy on the next package of material. So far the results suggest that problems with large addends might be better suited to induce spontaneous shortcut discovery and/or usage as compared to small addends. As a potential account for such an effect, several authors reported that participants were more likely to adopt strategies that have a significant performance advantage [42]. *Using* rather than just spotting a shortcut option in the first portion of material offered, in turn, might lead to better transfer to a different commutativity-based shortcut strategy later on, as a shortcut option that was once discovered but then not used might soon be forgotten. While there is evidence for that task difficulty can increase the likelihood that an already discovered shortcut is applied [50,51], it is also plausible, that extra effort is invested in searching for shortcut options in the face of relatively difficult problems.

Teaching should highlight relations among problems and should reinforce core concepts for example by special order practice problems [38]. Encouraging children’s understanding of the relations between different strategies will be helpful in order to promote the development of integrated concepts which in turn will help them to spontaneously use adaptive strategies. It might be promising to tackle the problem from both the conceptual and the procedural side, along the lines explored in the current work. For instance, recent work by Prather [52] suggests that interventions to increase conceptual understanding might be scaffolded by first strengthening procedural knowledge about a mathematical principle via implicit learning.

## Supporting Information

Figure S1 **Strategy benefit correlated with general arithmetic capability.** Cross-correlations between (a) absolute or relative benefit on either the addends-compare booklets or the ten-strategy booklets and (b) the independent baseline. Correlations with *p*< .05 are marked with an asterisks.
(TIF)

Table S1 **Time per problem and error rates per problem analyzed for booklet type and grade.**
(DOCX)

Table S2 **Error rates per problem type in the eyetracking study.**
(DOCX)

Text S1 Supporting Information.
(DOC)
