# Supplementary figures and images for: Spontaneous Usage of Different Shortcuts Based on the Commutativity Principle
Source: PLoS One. 2013 Sep 23;8(9):e74972. doi: 10.1371/journal.pone.0074972 (PMC3781138; doi:10.1371/journal.pone.0074972)

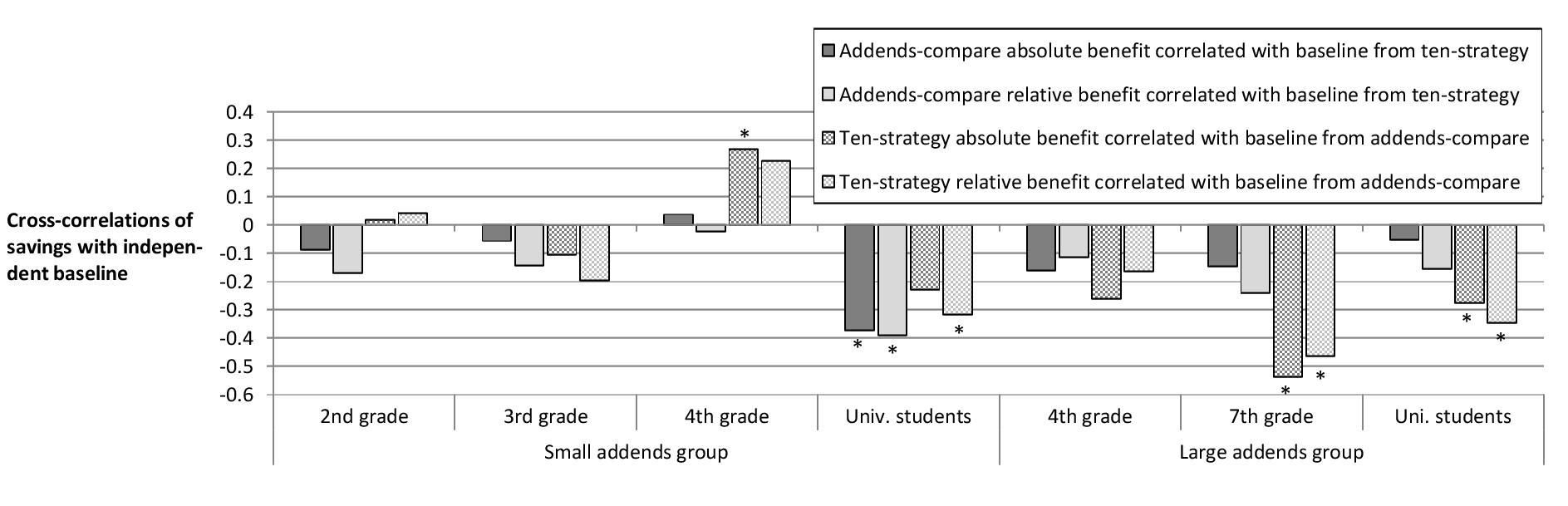

Supplement: Figure S1 — Strategy benefit correlated with general arithmetic capability. Cross-correlations between (a) absolute or relative benefit on either the addends-compare booklets or the ten-strategy booklets and (b) the independent baseline. Correlations with p< .05 are marked with an asterisks. (TIF) [file pone.0074972.s001.tif]
