# Supplementary material for: Spontaneous Usage of Different Shortcuts Based on the Commutativity Principle
Source: PLoS One. 2013 Sep 23;8(9):e74972. doi: 10.1371/journal.pone.0074972 (PMC3781138; doi:10.1371/journal.pone.0074972)
Supplement: Table S1 — Time per problem and error rates per problem analyzed for booklet type and grade. (DOCX) [file pone.0074972.s002.docx]

Table S1

Time per problem and error rates per problem analyzed for booklet type and grade

|  |  |  | | | Solution time in seconds | | | | | |
| --- | --- | --- | --- | --- | --- | --- | --- | --- | --- | --- |
|  |  | | Baseline | Addends- compare | | *p* | Baseline | Ten- strategy | *p* |  |
| Small addends | 2nd grade | | 19.55 | 17.65 | | .001 | 17.1 | 17.13 | .971 |  |
|  | 3rd grade | | 10.16 | 9.77 | | .186 | 8.33 | 7.37 | .017 |  |
|  | 4th grade | | 8.52 | 7,91 | | .005 | 8.80 | 8.41 | .119 |  |
|  | Univ. students | | 3.32 | 3.15 | | .029 | 2.71 | 2.34 | .001 |  |
| Large addends | 4th grade | | 19.23 | 16.04 | | .005 | 5.87 | 5.59 | .177 |  |
|  | 7th grade | | 16.28 | 14.16 | | .031 | 4.81 | 4.11 | .015 |  |
|  | Univ. students | | 8.93 | 8.06 | | .001 | 2.89 | 2.59 | .001 |  |

|  |  |  | | | % errors | | | | | |
| --- | --- | --- | --- | --- | --- | --- | --- | --- | --- | --- |
|  |  | | Baseline | Addends- compare | | *p* | Baseline | Ten- strategy | *p* |  |
| Small addends | 2nd grade | | 14.45 | 12.78 | | .232 | 9.34 | 10.87 | .381 |  |
|  | 3rd grade | | 5.58 | 10.35 | | .007 | 4.58 | 5.25 | .653 |  |
|  | 4th grade | | 4.63 | 6.23 | | .094 | 5.1 | 5.49 | .751 |  |
|  | Univ. students | | 3.91 | 4.73 | | .291 | 3.47 | 1.37 | .036 |  |
| Large addends | 4th grade | | 16.1 | 12.86 | | .159 | 5.62 | 1.01 | .001 |  |
|  | 7th grade | | 15.24 | 18.76 | | .156 | 5.65 | 3.22 | .108 |  |
|  | Univ. students | | 10.91 | 11.14 | | .884 | 5.44 | 1.75 | .01 |  |
