# Supplementary material for: Spontaneous Usage of Different Shortcuts Based on the Commutativity Principle
Source: PLoS One. 2013 Sep 23;8(9):e74972. doi: 10.1371/journal.pone.0074972 (PMC3781138; doi:10.1371/journal.pone.0074972)
Supplement: Table S2 — Error rates per problem type in the eyetracking study. (DOCX) [file pone.0074972.s003.docx]

Table S2

Error rates per problem type in the eyetracking study.

|  | % errors | |
| --- | --- | --- |
|  | Spontaneous | Instructed |
| Baseline booklet | 15.70 | 12.50 |
| Before addends-compare | 20.30 | 6.82 |
| Addends-compare | 12.50 | 5.68 |
| Baseline ten-strategy | 15.10 |  |
| Ten-strategy | 16.29 |  |
