# Supplementary material for: Spontaneous Usage of Different Shortcuts Based on the Commutativity Principle
Source: PLoS One. 2013 Sep 23;8(9):e74972. doi: 10.1371/journal.pone.0074972 (PMC3781138; doi:10.1371/journal.pone.0074972)
Supplement: Text S1 — Supporting Information. (DOC) [file pone.0074972.s004.doc]

**Supporting Information**

**Spontaneous usage of different shortcuts based on the commutativity principle**

Robert Gaschler, Bianca Vaterrodt, Peter A. Frensch, Alexandra Eichler, and Hilde Haider

**Additional Analyses –
Larger Shortcut Benefit for Children Calculating Fewer Problems**

We explored how the benefit taken from the addends-compare booklets as compared to the baseline booklets was related to calculation performance on booklets that did not offer shortcut options. This is relevant, to judge the proposal that a general mathematics ability rather than knowledge specific to the commutativity principle might to some extent explain the correlation of the strategy indicators. Assume that some students are better than others in many parts of procedural and conceptual knowledge about maths. They would calculate fast and would have a high probability to discover and use shortcut options. This would lead to a situation were any shortcut usage would correlate (not just those based on the same mathematical principle). Within the current study we therefore asked whether participants who are fast on baseline booklets would indeed show a large benefit on booklets with shortcut option. Correlation between (a) either relative or absolute benefit on the addends-compare booklet as compared to baseline and (b) the number of tasks solved on the baseline booklet was negative for all grader subsamples (all correlations *p*<. 05, range -.29 to -.58). Similarly, when correlating (a) either the relative or the absolute benefit on ten-strategy booklets with (b) the number of problems solved on the respective baseline booklets, all correlations were negative (range -.16 to -.63) and significant (except for fourth graders working on small addends). In either case, this should however in part be due to the dependence of the measures and regression towards the mean. In a more robust version, we correlated the benefit on booklets with shortcut option with the number of tasks solved from the *other* baseline booklet. That is, when targeting the benefit on addends-compare booklets we used the baseline booklet that had accompanied the ten-strategy booklet. When targeting the ten-strategy benefit we used the baseline booklet that went with the addends-compare booklet. These cross correlations were in most cases small and not significant, but most of them pointed in the negative direction (Figure S1). Thus, this analysis suggests, that the participants who were fast on baseline booklets in tendency profited less from the shortcut options. There was no indication that a general mathematical ability might explain the correlation between the two shortcut strategies.
